# Comparison of the diagnostic accuracy of commercial NS1-based diagnostic tests for early dengue infection

**DOI:** 10.1186/1743-422X-7-361

**Published:** 2010-12-06

**Authors:** Lyda Osorio, Meleny Ramirez, Anilza Bonelo, Luis A Villar, Beatriz Parra

**Affiliations:** 1 Grupo de Epidemiologia y Salud Poblacional (GESP) Escuela de Salud Publica, Facultad de Salud, Universidad del Valle. Cali, Colombia Calle 4b 36-140, Cali-Colombia; 2 Grupo de investigación VIREM, Escuela de Ciencias Basicas, Facultad de Salud, Universidad del Valle. Cali, Colombia. Calle 4b 36-140, Cali-Colombia; 3 Centro de Investigaciones Epidemiológicas y Centro de Investigaciones en Enfermedades Tropicales, Escuela de Medicina, Universidad Industrial de Santander, Sede Guatiguará Km 2 Autopista Piedecuesta, Santander, Colombia

## Abstract

**Background:**

We compared the diagnostic accuracy and reproducibility of commercially available NS1-based dengue tests and explored factors influencing their sensitivities.

**Methods:**

Paired analysis of 310 samples previously characterized as positive (n = 218) and negative (n = 92) for viral isolation and/or RT-PCR and/or IgM seroconversion. Masked samples were tested by two observers with Platelia™ Dengue NS1 Ag, second generation Pan-E™ Dengue Early ELISA, SD Dengue NS1 Ag ELISA, Dengue NS1 Ag STRIP™, and SD BIOLINE™ Dengue Duo (NS1/IgM/IgG).

**Results:**

SD BIOLINE™ NS1/IgM/IgG had the highest sensitivity (80.7% 95%CI 75-85.7) with likelihood ratios of 7.4 (95%CI 4.1-13.8) and 0.21 (95%CI 0.16-0.28). The ELISA-format tests showed comparable sensitivities; all below 75%. STRIP™ and SD NS1 had even lower sensitivities (<65%). The sensitivities significantly decreased in samples taken after 3 days of fever onset, in secondary infections, viral serotypes 2 and 4, and severe dengue. Adding IgM or IgG to SD NS1 increased its sensitivity in all these situations.

**Conclusions:**

The simultaneous detection of NS1/IgM/IgG would be potentially useful for dengue diagnosis in both endemic and non endemic areas. A negative result does not rule out dengue. Further studies are required to assess the performance and impact of early laboratory diagnosis of dengue in the routine clinical setting.

## Background

Dengue is a vector borne disease rapidly spreading in urban areas in tropical and subtropical countries. It is estimated that at least 10% of dengue fever cases evolve to severe and eventually lethal forms of the disease. The clinical and laboratory findings in dengue are very similar to those of other febrile diseases that are prevalent in the same geographical regions [1]. Therefore, a dengue diagnostic test is required for adequate case management and to reduce misclassification in the dengue surveillance system. However, dengue diagnosis in the first days of fever is yet problematic.

There are three main laboratory methods to diagnose dengue infection: viral isolation in culture, detection of viral RNA, and specific IgM/IgG antibodies in paired sera. The gold standard is usually a combination of these methods [1,2]. Viral isolation is costly, the results are usually available after 6 to 10 days and it is only obtainable in laboratories with the appropriate infrastructure for cell culture or mosquito colonies. The RT-PCR and other PCR-based techniques give results within 24 hours but they are also costly and they are not available for most clinicians. On the contrary, there are commercially available immunochromatographic and ELISA tests for the detection of IgM/IgG antibodies which give results within minutes or few hours. However, the detection of antibodies in a dengue infected person is only possible after 4-5 days of disease onset. Moreover, a single positive IgM or IgG result suggests recent infection but paired sera samples showing seroconversion or a fourfold titer increase are required to confirm diagnosis [1].

Recently, several dengue diagnostic tests based on the detection of NS1 (Non-structural Protein 1) have become commercially available. NS1 is a highly conserved glycoprotein of flaviviruses including Dengue, Japanese encephalitis, Yellow fever and tick-borne encephalitis virus [3]. The specificity of the NS1-based Dengue tests is reported to be between 86.1% and 100% and false positives are considered rare [4,5]. Higher variability (between 37% and 98.9%) has been reported in the sensitivity of these tests (Table 1) [6-24]. This variability could be partly explained by the fact that sensitivity has been found to decrease with time after fever onset and in secondary infections [12,18,21]. The addition of IgM and IgG specific antibodies detection to NS1-based tests in a single kit has been suggested [25] may improve the assessment of dengue infection status and one such test (SD BIOLINE™ Dengue Duo) has become commercially available. With all these options in the market, it is necessary to identify which of the current NS1-based diagnostic tests would be potentially more useful in the clinical setting. We sought to compare the performance of the current commercially available NS1-based assays for the early diagnosis (within 7 days since fever onset) of dengue infections. The objectives of this study were: 1) To identify differences in sensitivity, specificity, and likelihood ratios between all the diagnostic assays, 2) To describe the effect of duration of symptoms, type of infection, viral serotype, and severity of the disease on the sensitivity of the tests, and 3) to determine the reproducibility of each diagnostic test.

**Table 1:** Reported sensitivity and specificity of commercially available NS1-based dengue diagnostic tests

Test	Sensitivity % (95%IC)	Specificity % (95%CI)	place
STRIP™	61.6 (55.2-67.8)	100 (93.8-100)	Vietnam [6]
PanE™	49.4 (38.5-60.4)	100 (92.1-100)	India [7]
STRIP™	78.9 (70-86.1)	99 (94.6-99.9)	Singapore [8]
PanE™	67 (57.3-75.7)	100 (96.4-100)	Singapore [8]
Platelia™	81.7 (73.1-88.4)	100 (96.4-100)	Singapore [8]
STRIP™	89.6 (84.7-93.2)	99.1 (96.9-99.9)	Brazil [9]
PanE™	72.3 (65.8-78.1)	100 (98.4-100)	Brazil [9]
Platelia™	83.6 (78.1-88.2)	98.7 (96.2-99.7)	Brazil [9]
Platelia™	67.3 (57.1-76.4)	-	Finland [10]
Platelia™	37	99.5	Vietnam [5]
Platelia™	73.6 (63.7-81.6	-	Australia [11]
PanE™	63.7 (53.5-72).	-	Australia [11]
Platelia™	83.2 (75.5-89.3)	100 (86.7-100.0)	Vietnam [12]
Platelia™	71.3 (61-80)	86.1 (70.9-94.4)	Venezuela [13]
Pan E™	60.9 (50.4-70.5)	94.4 (80.9-99.4)	Venezuela [13]
STRIP™	67.8 (57.4-76.7)	94.4 (80.9-99.4)	Venezuela [13]
STRIP™	90.4 (86.6-94.4)	99.5 (97.4-99.9)	Malaysia [14]
STRIP™	98.9 (96.8-100)	90.6 (85.6-95.7)	Thailand [15]
STRIP™	77.3 (0.54-0.92)	100	Taiwan [16]
Pan E™	83.3 (65.2-94.3)	-	China [17]
Platelia™	63.2 (55.7-70.0)	98.4 (91.7-99.7)	Thailand [18]
Platelia™	83.2 (77.5-87.7)	100 (92.1-100)	Puerto Rico [19]
PanE™	64.9 (58.2-71.1)	97.8 (88.4-99.6)	Puerto Rico [19]
STRIP™	77.6 (72.1-82.4)	100 (92.6-100)	French Guiana [20]
Platelia™	82.4 (77.3-86.7)	100 (92.6-100)	French Guiana [20]
Pan E™	55.1 (49.0-61.2)	97.9 (88.9-99.9)	French Guiana [20]
Platelia™	92.3 (64-99.8)	100	Thailand [21]
PanE™	63 (53-73)	100	Laos [22]
Platelia™	93.4 (89.2-96.3)	100 (98.9-100)	Singapore [23]
Platelia™	88.7 (94-92.4)	100 (98.9-100)	French Guiana [24]

## Methods

### Type of study and sample size calculation

The study was a cross sectional case-reference design to assess diagnostic tests [26]. A paired analysis of samples from febrile subjects with and without dengue was done using viral isolation, RT-PCR or IgM seroconversion as gold standard. Sample size for dengue (n = 210) and non-dengue (n = 100) was estimated based on an expected 90% sensitivity and 100% specificity for the Platelia™ test versus 80% sensitivity and 90% specificity for the other assays. The Conner method for the paired McNemar test was used for sample size calculation with a 5% alfa and 20% beta errors [27]. Half dengue and no dengue samples were used to assess reproducibility.

### Clinical samples

Stored serum (229, 73.9%) or plasma (81, 26.1%) samples from febrile subjects with clinically-suspected dengue infection who took part in studies carried out by Universidad del Valle and Universidad Industrial de Santander in Colombia between 2004 and 2008 were selected randomly. The following criteria were considered: 1) dengue status known as a result of one or more of the following: viral isolation, RT-PCR or IgM seroconversion, 2) sample taken between day 0 and 7 of onset of fever, and 3) a minimum of 1 mL volume available. Day 0 was defined as the same day of fever onset. To avoid the spectrum bias, samples representing subjects who had been previously classified as dengue fever and hemorrhagic dengue were included and further classified as non-severe and severe dengue, respectively [1].

Gold standard tests (viral culture, nested RT-PCR or paired IgM) had been done during the previous studies at the virology laboratory of Universidad del Valle. Briefly, for viral isolation sera samples had been cultured in the mosquito cell line clone C6/36 HT and incubated at 33°C for 10 to 14 days. Viruses were detected and identified by immunofluoresce with serotype specific monoclonal antibodies 15F3 (DENV1), 3H5 (DENV2), 5D4 (DENV3) and 1H10 (DENV4) (Chemicon International, Inc. Temecula, California) and fluorescein isothiocyanate-conjugated goat anti-mouse antibody [28]. For RT-PCR, viral RNA had been extracted with trizol (Gibco-BRL, Gaithersburg, MD) and cDNA obtained with the reverse transcriptase of the Avian myeloblastosis virus (Promega, Madison, WI) and a dengue universal antisense primer targeting the C/prM region of the genome. cDNA amplification was performed with a nested PCR using the same universal dengue primers in a first round of amplification and viral serotype specific primers in a second round of PCR [29]. Finally, IgM MAC-ELISA in paired samples had been done using affinity-purified goat anti-human IgM as a capture antibody (KPL; Gaitersburg, Maryland 1 μg/ml), followed by addition of 1:40 dilution of serum samples duplicates. Assay antigen was home-made and consisted of a mixture of 4 HA U (hemoagglutinating units) each of the four dengue serotypes obtained by i.c. inoculation of suckling mice and antigen extraction by a sucrose/acetone gradient. Detection was performed using 1:10,000 dilution of a peroxidase-conjugated dengue-complex specific monoclonal antibody MAB 6B6C-1 (kindly provided by CDC, San Juan de Puerto Rico) and substrate p-nitrophenyl-phosphate [30,31]. Positivity was defined as having an assay absorbance of ≥2.0 (405 nm) after subtracting the background value (negative sample).

**Figure 1:**
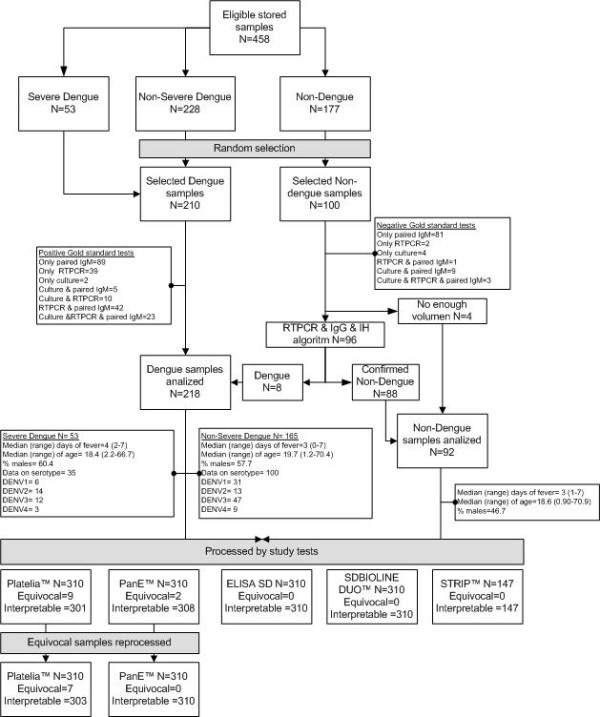
**Study sample selection and laboratory analyses**.

Because up to 30% of secondary dengue infections do not have detectable IgM [32], and most non-dengue samples had been analyzed only by paired IgM, samples classified as non-dengue were further analyzed using an algorithm of RT-PCR plus IgG and IH. All non-dengue samples (except for four samples with insufficient volume left) were processed with RT-PCR as described elsewhere [29]. RT-PCR positives were considered as dengue. To discard secondary infections which do not increased IgM, all RT-PCR negative non-dengue samples underwent IgG detection in acute sera using Dengue Duo (IgM/IgG) Cassette (Inverness - Brisbane, Australia). Non-dengue samples with negative IgG were considered as true negatives. Those samples with positive IgG were processed with haemagglutination-inhibition test (HI) and considered as true negatives if not increased (>2560) titers were detected in convalescent sera (Figure 1). HI was done at the virology laboratory of Universidad del Valle using goose red blood cells and sucrose/acetone extracted antigens obtained in suckling mice brains following Kuno et al. 1991 [33]. This study was approved by the Universidad del Valle Ethics Review Board.

### Diagnostic tests and procedures

All 5 diagnostic NS1-based tests commercially available at the time of the study were analyzed. These included: Platelia™ Dengue NS1 Ag Test (Bio-Rad Laboratories - Marnes La Coquette, France), second generation Pan-E™ Dengue Early ELISA (Inverness - Brisbane, Australia), Dengue NS1 Ag ELISA (Standard diagnostic Inc. - Kyonggi-do - South Korea), Dengue NS1 Ag STRIP™ (Bio-Rad), and SD BIOLINE™ Dengue Duo (Standard diagnostic Inc.). The characteristics of the tests are summarized in table 2. The Platelia™ Dengue NS1 Ag Test and Dengue NS1 Ag STRIP™ were purchased from the local distributor while the rest were kindly donated by the manufacturers. All tests were run following the corresponding manufacturer's instructions. Dengue NS1 Ag STRIP™ was read at 15 min and 30 min. Three separate results were obtained from SD BIOLINE™ Dengue Duo test based on the results of NS1 only (dengue if NS1 was positive and non-dengue if NS1 was negative, regardless of IgM/IgG results), NS1/IgM combined (dengue if one of NS1 or IgM was positive and non-dengue if both were negative, regardless of IgG results), and NS1/IgM/IgG combined (dengue if at least one of NS1, IgM or IgG was positive and non-dengue if all three were negative). Batches of samples were analyzed by all the NS1-based diagnostic tests on the same day and by the same persons who were two experienced lab scientists. Both observers were blind to the samples dengue status and each other results. Results of the ELISA-based format tests given as "equivocal" were repeated once. Persistent equivocal results were excluded from the analysis. Those results of the immunochromatography-based format tests given as "weak" were considered as positive results.

**Table 2:** Characteristics of the evaluated NS1-based dengue diagnostic tests

Test name	Platelia™ Dengue NS1 Ag Test	Pan-E™ Dengue Early	SD Dengue NS1 Ag	Dengue NS1 Ag STRIP™	SD BIOLINE™ Dengue Duo test
**Company**	Bio-Rad	Inverness	Standard diagnostic Inc	Bio-Rad	Standard diagnostic Inc
					
**Country**	France	Australia	South Korea	France	South Korea
					
**Method of detection**	ELISA	ELISA	ELISA	Immunochromatography	Immunochromatography
					
**Number of tests per pack**	96	96	96	25	1 (of each NS1, IgM and IgG)
					
**Volume of sample required**	50 μL	60-75 μL	50 μL	50 μL	85 μL (75 for NS1 plus 10 for IgM/IgG)
					
**Time to result**	2.5 h	2.5 h	2.5 h	15-30 min	20 min
					
**Additional equipment required?**	Yes	Yes	Yes	No	No

### Statistical analysis

Data were double entered and validated using Epinfo (Centers for Disease Control and Prevention, USA, 2000). Stata 10 (Stata Corporation, 2003) was used for statistical analyses. First observer results were used to obtain sensitivity, specificity, negative (NPV) and positive (PPV) predictive values, positive and negative likelihood ratios (LR) with their corresponding 95% confidence intervals. Cochrane Q was used to compare overall performance of ELISA tests and of immunocromatographic tests. McNemar Chi squared test or the equivalent exact test was used to compare the diagnostic accuracy among each possible pair of assays. The method proposed by Roldan-Nofuentes and Del Castillo (2007) was used to identify significant statistical differences in the LR of all tests [34] and carried out in Mathematica 7 (Wolfram Research Inc., 2010). Sensitivities with their corresponding 95% confidence intervals were also calculated by stratum of duration of symptoms (≤3 and 4-7 days), primary/secondary infection (defined as absence/presence of specific IgG in acute sera based on the results of the SD Bioline™ Dengue Duo), severe and non severe infection, and viral serotype. Reproducibility of the tests (inter-observer agreement) was assessed using Kappa indexes (k). We interpreted k results as follows: values of less than 0, poor; 0 to 0.2, slight; 0.2 to 0.4, fair agreement; 0.4 to 0.6, moderate agreement; 0.6 to 0.8, substantial agreement; and values of 0.8 to 1.0 almost perfect agreement [35]. Funds allowed us to purchase a limited number of Dengue NS1 Ag STRIP™ and hence results were available for 147 samples (104 dengue and 43 non-dengue). It was not possible to assess reproducibility of this test. A P value <5% was considered as statistically significant.

## Results

A total of 310 samples were included in the study from which 210 were classified as dengue and 100 as non-dengue. Eight samples initially classified as non-dengue based on IgM negative results in paired serum samples were RT-PCR-positive and hence were reclassified as dengue. Therefore, for the final analysis there were 218 dengue and 92 non-dengue cases. Samples represented all age groups and had a median of 3 days of fever onset. Nine samples analyzed by Platelia™ and 2 by Pan E™ gave equivocal results and were run twice. The second time, both Pan E™ and 2 Platelia™ results were negative while the other 7 (1 non-dengue and 6 dengue) remained equivocal and were excluded from the final analyses (Figure 1). Sixty four (29.4%) dengue samples were positive for IgG in the SD Bioline™ Dengue Duo and were considered as secondary infections. Secondary infections had a median of 4 (range 2-7) days of fever onset and dengue serotype was identified in 42 of these cases: 13 DENV1, 17 DENV2, 7 DENV3, and 5 DENV4.

Sensitivity and specificity of tests ranged from 51% to 80.7% and from 89.1% to 96.7%, respectively (Table 3). SD BIOLINE™ NS1/IgM/IgG had the highest sensitivity (80.7% 95%CI 75-85.7) followed by SD BIOLINE™ NS1/IgM (78.4% 95%CI 72.4-83.7) and Pan E™ (71.1% 95%CI 64.6-77). There were not statistically significant differences in the diagnostic accuracy of the three ELISA-based assays (p = 0.9). For the immunocromatographic tests, STRIP™ read at 30 min had a higher diagnostic accuracy (71.4%, 105/147) than STRIP™ read at 15 min (68.7%, 101/147) but this difference was not statistically significant (p = 0.1). The diagnostic accuracy did not differ among SD BIOLINE™ NS1/IgM (82.2%, 255/310) and SD BIOLINE™ NS1/IgM/IgG 83.2, 258/310 (p = 0.4). However, their diagnostic accuracy was higher than all the other immunochromatographic and ELISA tests (p<0.05 for all pair wise comparisons).

**Table 3:** Measures of performance of commercially available NS1 detection-based dengue diagnostic assays

Assay	Dengue samples	Non-dengue samples	Positive tests	Negative tests	Sensitivity % (95% CI)	Specificity % (95% CI)	PPV % (95% CI)	NPV % (95% CI)	LR+ (95% CI)	LR- (95% CI)
**Platelia™**	212	91	150	84	70.8	92.3	95.5	57.5	9.2	0.31
					(64.1-76.8)	(84.8-96.9)	(91-98.2)	(49.1-65.7)	(4.5-18.8)	(0.25-0.39)
										
**PanE™**	218	92	155	82	71.1	89.1	94	56.6	6.54	0.32
					(64.6-77)	(80.9-94.7)	(89.1-97.1)	(48.1-64.8)	(3.62-11.8)	(0.26-0.4)
										
**ELISA SD™**	218	92	150	87	68.8	94.6	96.8	56.1	12.7	0.33
					(62.2-75)	(87.8-98.2)	(92.6-99)	(48-64.1)	(5.37-29.8)	(0.27-0.4)
										
**SD Bioline™**	218	92	111	89	51	96.7	97.4	45.4	15.6	0.5
**NS1 only**					(44.1-57.7)	(90.8-99.3)	(92.5-99.5)	(38.3-52.7)	(5.1-48)	(0.44-0.58)
										
**SD Bioline™**	218	92	171	84	78.4	91.3	95.5	64.1	9.02	0.23
**NS1/IgM**					(72.4-83.7)	(83.6-96.2)	(91.4-98.1)	(55.3-72.3)	(4.64-17.6)	(0.18-0.3)
										
**SD Bioline™**	218	92	176	82	80.7	89.1	94.6	66.1	7.43	0.21
**NS1/IgM/IgG**					(75-85.7)	(81-94.7)	(90.3-97.4)	(57.1-74.4)	(4.12-13.8)	(0.16-0.28)
										
**STRIP™**	104	43	60	41	57.7	95.3	96.8	48.2	12.4	0.44
**15 min**					(47.6-67.3)	(84.2-99.4)	(88.8-99.6)	(37.3-59.3)	(3.17-48.5)	(0.35-0.56)
										
**STRIP™**	104	43	64	41	61.5	93.3	97	50.6	13.2	0.4
**30 min**					(51.5-70.9)	(84.2-99.4)	(89.5-99.6)	(39.3-62)	(3.4-51.6)	(0.31-0.5)

PPV = Positive Predictive Value, NPV = Negative Predictive Value, LR = Likelihood Ratio

**Figure 2:**
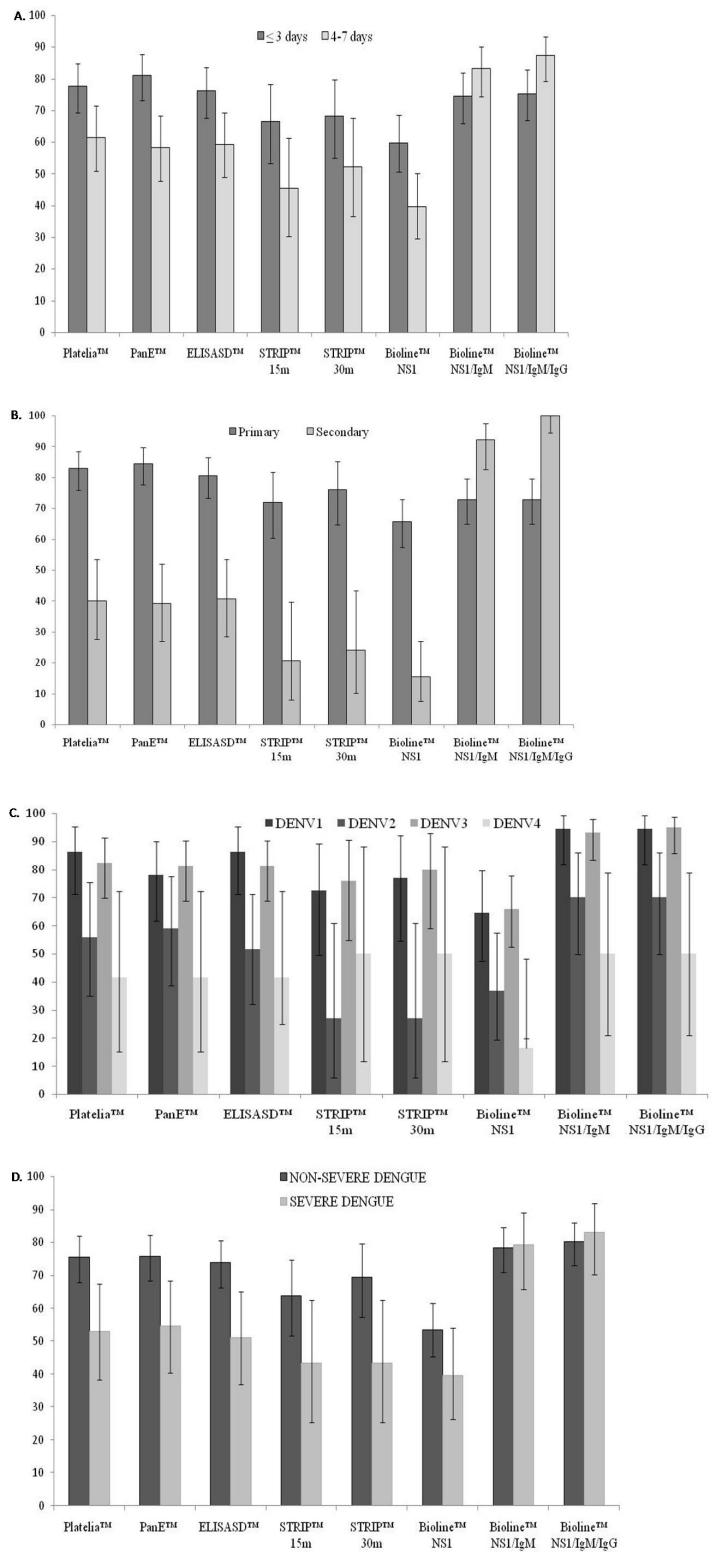
**Sensitivity (with corresponding 95%CI) of NS1-based dengue diagnostic tests according to days of fever onset (A), type of infection (B), viral serotype (C), and (D) severity of disease**.

In line with the relatively high specificity found, the PPVs were above 90% for all tests. In contrast, the highest NPV was 66.1% (95%CI 57.1-74.4). LR+ varied between 6.5 and 15.6 while LR-varied between 0.2 and 0.5 (Table 3). Statistically significant differences in LR were found between all tests pair wise comparisons except Platelia™ Vs. PanE™, Platelia™ Vs. STRIP™, and ELISA SD™ Vs. STRIP™. The sensitivity of NS1-based diagnostic tests significantly decreased in those samples taken after 3 days of fever onset, in secondary infections, viral serotypes 2 and 4, and severe dengue. Adding IgM or IgG to SD BIOLINE™ NS1 increased its sensitivity in all these situations (Figure 2). The positive effect of adding IgM to NS1 in the sensitivity of the test was more noticeable in samples with detectable IgG regardless of the days of fever onset (Table 4).

**Table 4:** Sensitivity of SD Bioline™ Dengue Duo according to presence of IgG and days of fever.

Component of SD Bioline™ Dengue Duo analyzed		Negative IgG in acute sera		Positive IgG in acute sera	
		
	Days of fever onset	0 to 3	4 to 7	0 to 3	4 to 7
	
	Dengue samples	N = 108	N = 46	N = 14	N = 50
Only NS1	Positive tests	71	30	2	8
	Sensitivity %	65.7	65.2	14.3	16
	(95% CI)	(56-74.6)	(49.8-78.6)	(1.78-42.8)	(7.17-29.1)
					
Any of NS1/IgM	Positive tests	78	34	13	46
	Sensitivity %	72.2	74	93	92
	(95% CI)	(62.8-80.4)	(59-85.7)	(66.1-99.8)	(80.8-97.8)
					
Any of NS1/IgM/IgG	Positive tests	78	34	14	50
	Sensitivity %	72.2	74	100	100
	(95% CI)	(62.8-80.4)	(59-85.7)	(76.8-100)	(92.9-100)

The inter-observer agreement was almost perfect for Pan E™ (k = 0.94 CI95% 0.88-0.99), ELISA SD™ (k = 0.89 CI95% 0.82-0.96), SD BIOLINE™ NS1 (k = 0.90 CI95% 0.83-0.98), and the IgM cassette of SD BIOLINE™ (k = 0.85 CI95% 0.76-0.94) while substantial agreement was observed with Platelia™ (k = 0.75 CI95% 0.60-0.89) and the IgG cassette of SD BIOLINE™ (k = 0.7 CI95% 0.56-0.84).

## Discussion

In the present study we compared simultaneously the performance of 5 commercially available tests for the early (within 7 days of fever onset) diagnosis of dengue. The sensitivity (51% to 80.7%) and specificity (89.1% to 96.7%) of the NS1-based tests found in the present study fell within the range described elsewhere (Table 1). In previous comparative studies the sensitivity of Platelia™ (71.3%-87.4%) was consistently higher than STRIP™ (67.8%-82.4%) and, in turn, the sensitivity of STRIP™ was higher than Pan E™ (60.4%-64.9%). By contrast, we did not find differences in the diagnostic accuracy of the ELISA-format diagnostic tests (Platelia™, Pan E™ and ELISA SD™). In the present study, Pan E™ sensitivity was higher than in the previous reports probably because we used Pan E™ second generation, which uses less diluted controls and samples (1:2 instead 1:10) than the previous version [36]. Despite ELISA-format tests showing comparable sensitivities, they were all below 75%. The immunocromatographic-format tests that detect only NS1 had even lower sensitivities. This means that a negative result on any of these tests does not rule out dengue. The immunocromatographic SD Bioline™ that detects simultaneously NS1 and specific IgM/IgG showed the highest sensitivity (80.7% CI95% 75-85.7) which was comparable to the 83.7% (95%CI 78.4 - 88.1) reported in Vietnam [6]. Similarly, the addition of IgM has shown to improve the sensitivity of NS1 ELISA-format tests from 63.2% to 79% on admission samples without significantly decreasing specificity [22]. Although we did not find statistically significant differences in the addition of IgM only or both antibodies to NS1, the use of IgG could have clinical significance when correlated with disease evolution and the days of fever onset. In any case, a positive result for IgM or IgG in a single sample does not confirm dengue, therefore; the impact of false positives in the routine clinical setting should be assessed.

Predictive values depend on the prevalence of the disease but their trend here showed that all tests were comparable. For potential clinical use, LR measures of diagnostic tests performance are more useful than predictive values. They tell how the test results modified the pretest probability of disease independent of its prevalence. LR values above 10 and below 0.1 are considered conclusive to rule in or rule out diagnoses, respectively while values of 5 to 10 and 0.1 to 0.2 are frequently helpful to take clinical decisions [37]. In a scenario where a clinician's interest is to confirm dengue diagnosis any of the tests is likely to be useful but they should be aware that a negative test does not rule out dengue. Hence, further diagnostics such as paired IgM or IgG to assess seroconversion or titer increase would need to be done. On the contrary, if ruling out dengue is important for clinical decision then the SD Bioline™ NS1/IgM/IgG would provide more useful information than any of the other tests (LR = 0.21). However, it ought not to be considered as a screening test. Further studies with larger sample size would be required to give more precise estimates of LR.

The simultaneous detection of NS1 and specific IgM/IgG appeared to overcome the limitations of using only NS1-based diagnostic tests in secondary infections, in subjects who seek treatment after 3 days of disease onset and on severe cases. Our results confirmed that sensitivity of NS1-based tests decreased in secondary infections and with time of fever onset [12,18,20,21,23]. In contrast to Chaiyaratana et al. (2009), we also found a decreased sensitivity of all assays in severe cases because, in our study, these were more likely to be secondary infections (OR = 3,01 95%CI 1.57-5.78) and presented after 4 days of fever (OR = 4.31 95%CI 2.04-9.1) than non-severe infections [15]. The addition of IgM/IgG appeared to increase the sensitivity of NS1 Bioline™ in all 4 dengue serotypes. Nevertheless, the sensitivities of all tests were consistently lower in DENV2 and DENV4 infections as was also observed in Venezuela [13] and Puerto Rico [19]. The frequency of secondary infections and time since disease onset could only partly explain these differences because, in our study, only DENV4 tended to be associated with the presence of IgG in acute sera and samples taken after 4 days of fever (data not shown). Viraemia levels were found to be significantly higher in DENV1 than DENV2 and DENV4 infected subjects in Vietnam [38]. Therefore, differences in viral loads could be an alternative explanation for the observed effect of serotype in NS1-based tests sensitivity. In spite of this, no differences in sensitivity of Platelia™ and STRIP™ according to serotype had been reported in samples from French Guiana [20,24]. Therefore, other reasons such as a decreased avidity of NS1 tests for certain geographical dengue virus clades, as proposed before, could be explored [13].

One limitation of the present study was residual misclassification of non-dengue cases, which would underestimate the specificity of the tests. This is probably low because most negative (92/98) samples were analyzed by at least two gold standard methods. Confirmation of non-dengue diagnosis was not sought due to limited sample volume. Specificities were relatively high and similar to previous reports in South America [13]. Further misclassification is likely in secondary and primary infections as we did not use a gold standard method such as a quantitative HI due to limited funds and in some samples not enough available volume. The use of one of the study tests (SD Bioline™) to define presence of both IgG and IgM would tend to overestimate the sensitivity of IgM in secondary infections. Nevertheless, the findings were consistent with previous reports of the increased test sensitivity in secondary infections provided by the addition of IgM to NS1 [6]. Yellow Fever vaccination (YFV) also is a source of potential false positives with IgG but this information was not available for the study samples. YFV is recommended in Colombia and therefore it is important to include information on YFV status of subjects in future studies to assess the degree of misclassification.

## Conclusions

Of the 5 tests assessed, SD Bioline™ NS1/IgM/IgG performed significantly better than the other tests. Therefore, the simultaneous detection of NS1/IgM/IgG would be potentially useful to diagnose dengue in both endemic and non endemic areas. All NS1 tests were highly reproducible. Clinicians must be aware that a negative result does not rule out dengue. To take evidence based decisions about the usefulness of this test in clinical settings, it is recommended to assess its performance in consecutive subjects with potential dengue infection under routine conditions at health centers with different levels of complexity. Further studies are required to assess the potential impact of implementing early laboratory diagnosis of dengue in terms of prognosis and cost-effectiveness. Secondary infection, viral serotype and time since fever onset should be taken into account as sources of heterogeneity in the interpretation and meta-analysis of NS1-based diagnostic tests.

## Competing interests

None of the authors have received any gifts, travel funds, have served as consultant, speaker or received previous funding from the diagnostic tests manufacturers. The present study was partially funded by Standard Diagnostics Inc. However, none of the diagnostic test manufacturers had a role, either directly or through a third party, in the gathering or preparation of data, in the writing of the manuscript or the decision to submit the manuscript for publication. There are not any other financial and non-financial competing interests.

## Authors' contributions

LO designed the study, conducted the data analysis and wrote the manuscript; MR designed the study, entered and analyzed the data and critically reviewed the manuscript; AB contributed to acquisition of data and interpretation of the results, run the tests as a second observer and critically reviewed the manuscript; LAV contributed to acquisition of data and interpretation of the results, and critically reviewed the manuscript; BP contributed to acquisition of data and interpretation of the results, run the tests as a first observer and co-wrote the manuscript. All authors read and approved the final manuscript.
